# Ru_3_@Mo_2_CO_2_ MXene single-cluster catalyst for highly efficient N_2_-to-NH_3_ conversion

**DOI:** 10.1093/nsr/nwae251

**Published:** 2024-07-26

**Authors:** Cong Zhang, Ze-Hui Wang, Haiyan Wang, Jin-Xia Liang, Chun Zhu, Jun Li

**Affiliations:** School of Chemistry and Chemical Engineering, Guizhou University, Guiyang 550025, China; Shaanxi Key Laboratory of Catalysis, Institute of Theoretical and Computational Chemistry, School of Chemistry and Environment Science, Shaanxi University of Technology, Hanzhong 723000, China; Department of Chemistry and Guangdong Provincial Key Laboratory of Catalytic Chemistry, Southern University of Science and Technology, Shenzhen 518055, China; School of Chemistry and Chemical Engineering, Guizhou University, Guiyang 550025, China; School of Chemistry and Chemical Engineering, Guizhou University, Guiyang 550025, China; School of Chemistry and Chemical Engineering, Guizhou University, Guiyang 550025, China; Department of Chemistry and Guangdong Provincial Key Laboratory of Catalytic Chemistry, Southern University of Science and Technology, Shenzhen 518055, China; Department of Chemistry and Guangdong Provincial Key Laboratory of Catalytic Chemistry, Southern University of Science and Technology, Shenzhen 518055, China; Department of Chemistry and Engineering Research Center of Advanced Rare-Earth Materials of Ministry of Education, Tsinghua University, Beijing 100084, China; Fundamental Science Center of Rare Earths, Ganjiang Innovation Academy, Chinese Academy of Sciences, Ganzhou 341000, China

**Keywords:** N_2_ reduction reaction, single-cluster catalyst, Ru_3_@Mo_2_CO_2_, DFT, *ab initio* molecular dynamics simulation

## Abstract

Single-cluster catalysts (SCCs) representing structurally well-defined metal clusters anchored on support tend to exhibit tunable catalytic performance for complex redox reactions in heterogeneous catalysis. Here we report a theoretical study on an SCC of Ru_3_@Mo_2_CO_2_ MXene for N_2_-to-NH_3_ thermal conversion. Our results show that Ru_3_@Mo_2_CO_2_ can effectively activate N_2_ and promotes its conversion to NH_3_ through an association mechanism, in which the rate-determining step of NH_2_* + H* → NH_3_* has a low energy barrier of 1.29 eV. Notably, with the assistance of Mo_2_CO_2_ support, the positively charged Ru_3_ cluster active site can effectively adsorb and activate N_2_, leading to 0.74 |e| charge transfer from Ru_3_@Mo_2_CO_2_ to the adsorbed N_2_. The supported Ru_3_ also acts as an electron reservoir to regulate the charge transfer for various intermediate steps of ammonia synthesis. Microkinetic analysis shows that the turnover frequency of the N_2_-to-NH_3_ conversion on Ru_3_@Mo_2_CO_2_ is as high as 1.45 × 10^−2^ s^−1^ site^−1^ at a selected thermodynamic condition of 48 bar and 700 K, the performance of which even surpasses that of the Ru B5 site and Fe_3_/θ-Al_2_O_3_(010) reported before. Our work provides a theoretical understanding of the high stability and catalytic mechanism of Ru_3_@Mo_2_CO_2_ and guidance for further designing and fabricating MXene-based metal SCCs for ammonia synthesis under mild conditions.

## Teaser

This study thsoretically predicts that MXene-based Ru_3_@Mo_2_CO_2_ can effectively activate N_2_ and promote its conversion to NH3 via an association mechanism.

## INTRODUCTION

Ammonia synthesis (N_2_ + 3H_2_ → 2NH_3_) is one of the most important processes for agriculture, industrial productions and energy resources [1–3]. Yet the large-scale production of ammonia relies on the Haber–Bosch process [4–6], which requires high temperature and pressure (typically ∼500°C and ∼200 bar) [7–9] to directly dissociate the chemisorbed N_2_ over the bulk surfaces of Fe- and Ru-based catalysts [10–15]. Due to its high bonding energy of 941 kJ/mol and high N_2_-ionization potential of 15.1 eV [16], industrial N_2_-to-NH_3_ conversion accounts for ∼2% of global energy consumption and a large amount of CO_2_ emissions [8,17]. Moreover, limited by the Brønsted–Evans–Polanyi (BEP) scaling relation [18], the activity of catalysts and the reaction rate are not compatible with effective N_2_-to-NH_3_ conversion at low temperature. Here the BEP linear relationship between the activation energy and the enthalpy change of an elementary reaction regulates the dissociation barrier of N_2_ and the desorption energies of NH*_x_* that scale linearly with the adsorption energy of an N atom. Therefore, it is ideal to develop highly active catalysts for N_2_ fixation and activation under mild conditions.

Similar to single-atom catalysts (SACs, such as Pt_1_/FeO*_x_* [19]), which represent atomically precise heterogeneous catalysts featuring a substrate-anchored, stable and reactive (metal or non-metal) single-atom-based active center toward selective catalytic conversion of chemical compounds [20], single-cluster catalysts (SCCs) [21–25] provide tunable and atomically precise catalytic active sites for handling complicated redox reactions in heterogeneous catalysis. The supported single clusters of SCCs can provide multiple-atom active sites that show a synergistic effect for efficiently catalyzing complex reactions [21,26,27]. It is well documented that transition-metal single clusters anchored on suitable substrates can enhance the catalytic activity for N_2_-to-NH_3_ conversion, or even break the constraint of BEP scaling relation through the association mechanism involving the gradual hydrogenation of N≡N followed by the N–N cleavage for N_2_-to-NH_3_ conversion [22,23,28–30]. Our recent theoretical studies also showed that the synergistic effect of small metal single clusters of SCCs, such as Fe_3_ on θ−Al_2_O_3_ (010) surface [23] and Rh_1_Co_3_ on CoO (011) surface [22], could effectively catalyze NH_3_ synthesis at multiple-atom sites under relatively low temperature.

Currently, noble metal Ru-based catalysts are the second generation for ammonia synthesis due to their high activity under low temperature and pressure conditions [3,31,32]. A number of studies have shown that the geometric and electronic structures of the Ru active sites of Ru-based catalysts are sensitive to N_2_ activation for NH_3_ synthesis [33–39]. Moreover, the synergistic effect between the surface species and the supported Ru clusters, and the size effect of Ru-based catalysts, can facilitate thermal N_2_-to-NH_3_ conversion under mild conditions. For example, the synergism of the surface Sm−H species and Ru clusters on Ru/Sm_2_O_3_ SCCs [28] and the synergistic effect between the surface TiCN species and the supported Ru cluster on Ru/ZrH_2_ [40] can significantly improve their catalytic activity for NH_3_ synthesis from N_2_ under mild conditions. In addition, the small Ru size in Ru*_x_*/BaCeO_3_ is efficient with regard to NH_3_ synthesis as it enhances hydrogen spillover [36]. The size sensitivity of graphene-supported Ru catalysts from nanoparticles to subnanometric clusters and atomic clusters were also explored for ammonia synthesis experimentally and theoretically [41]. Therefore, it is crucial to explore the catalytic process and understand the mechanism of Ru-based SCCs for thermal ammonia synthesis at mild conditions.

Catalytic properties of SCCs are determined by both the metal cluster and support. MXene, as a new class of 2D carbide, nitride and carbonitride of transition-metal nanomaterials, is a robust new type of support that can bind Ru clusters tightly. MXenes are derived from the MAX-phase precursors by etching the ‘A’ element, in which ‘M’ stands for early d-block transition metal element, ‘A’ for the main group's sp-block element, and ‘X’ for C and/or N atom [42–44]. Due to its high stability, extremely easily tunable atomic surface with various single-atom adsorption sites, outstanding electronic conductivity and excellent catalytic properties [45–47], MXene is a robust substrate for SACs. Furthermore, MXene can evolve into thermally stable M*_n_*_+1_X*_n_*T*_x_* by bonding M*_n_*_+1_X*_n_* with terminal atoms T (T = O, F, S, OH, etc.) [48–51], thus further expanding adsorption and doping sites for single atoms (SAs) on its surface. Based on its structural features, a large number of MXene-based metal and non-metal [52] SACs have been studied theoretically and/or experimentally [53,54]. Tao *et al.* reported that by adjusting the annealing temperature, transition metal Ru clusters of different sizes can be obtained on MXene Ti_3_C_2_T*_x_* catalysts, indicating that it is feasible to load clusters of specific sizes on MXene [55]. Additionally, a large number of studies have shown that MXenes can effectively adsorb, activate or dissociate N_2_ [56–58]. Recently, a Mo_2_CO_2_ MXene-supported catalyst with highly dispersed Ru clusters was successfully synthesized [59]. The interplay of MXenes and Ru nanoparticles can further promote catalytic efficiency in ammonia synthesis [60]. Tan *et al.* reported that the single-atom Ru-modified Mo_2_CO_2_ MXene exhibits excellent electrocatalytic performance for nitrogen fixation [61].

We have therefore chosen 2D O-functional Mo_2_C (Mo_2_CO_2_) MXene as the support to anchor the small Ru_3_ cluster to build the Ru-based Ru_3_@Mo_2_CO_2_ SCC, taking advantage of its chemical stability [62,63] and controllable sites [64] to stably anchor SAs or single clusters [42]. The triatomic Ru_3_ cluster is particularly stable due to effective metal–metal bonding when compared with Ru_2_ and Ru_4_ clusters, as shown in the case of an M*_x_* cluster supported on graphydiyne [65]. *Ab-initio* molecular dynamics (AIMD) simulations revealed that an Ru_3_ cluster could be stably anchored on the surface of Mo_2_CO_2_ MXene by bonding with three surface O atoms. Based on the stable structure of Ru_3_@Mo_2_CO_2_, the optimal association mechanism for thermal N_2_-to-NH_3_ conversion at low temperature was explored. A series of theoretical analysis methods were performed to explore the catalytic properties of Ru_3_@Mo_2_CO_2_ for NH_3_ synthesis. Finally, the microkinetic simulations predicted the turn-over frequencies (TOFs) through different reaction paths for ammonia synthesis catalyzed by Ru_3_@Mo_2_CO_2_.

## COMPUTATIONAL DETAILS

The geometries optimization and electronic structure calculations were performed with spin-polarized density functional theory (DFT) using the Vienna *ab initio* simulation package (VASP) [66,67]. A generalized gradient approximation (GGA) with the Perdew–Burke–Ernzerhof (PBE) functional was used to describe the electronic exchange-correlation potential [68]. A cut-off energy of 450 eV was used for the plane-wave basis sets. A conjugated gradient method with a converging tolerance of 0.02 eV/Å for the force on each atom was used for the full geometry optimization without any restrictions. A vacuum distance of 15 Å was set to avoid artificial interlay interaction between the adjacent units of the periodic 2D materials. The van der Waals interaction was taken into account using the dispersion correction of Grimme's method (DFT-D3) [69,70]. A 3 × 3 × 1 Monkhorst-Pack grid was adopted for geometry optimization, while a 11 × 11 × 1 Monkhorst-Pack grid was used for the density-of-states (DOS) calculations. An integrated crystal orbital Hamilton population (ICOHP) was used to analyze the bonding/antibonding population between the adsorbates and the substrate Mo_2_CO_2_ [71,72]. The transition states were searched using the dimer method [73] and further confirmed by vibrational frequency analysis. Moreover, the stability and the adsorption behavior of Ru_3_ cluster anchored on the surface of Mo_2_CO_2_ were estimated by using AIMD simulations in a canonical ensemble (NVT) at 473 and 673 K for 20 ps with a time step of 1 fs.

Microkinetic simulations were performed to elucidate the TOFs and the conversions of ammonia synthesis on Ru_3_@Mo_2_CO_2_ using CatMAP software [74]. For surface reactions, the rate constants for the forward and backward elementary reaction were determined by the Eyring equation [75]. The harmonic approximation that treats all degrees of freedom as vibrational modes can be used to estimate the free energies of adsorbates at different temperatures. Detailed descriptions of microkinetic simulation methods are provided in the literature [76–78].

The quantum theory of atom-in-molecule (QTAIM) [79] was used to calculate the atomic Bader charges. This has been shown to provide intriguing bonding and electronic properties of SACs [80]. Following the convention of thermodynamics, the adsorption energy (${{E}_{{\mathrm{ads}}}}$) of an adsorbate (X) on the adsorbent Ru_3_@Mo_2_CO_2_ was defined by equation (1)


(1) \begin{eqnarray*}
{{E}_{{\mathrm{ads}}}}{\mathrm{\ = \ }}{{E}_{{\mathrm{X \ldots R}}{{{\mathrm{u}}}_{\mathrm{3}}}{\mathrm{@M}}{{{\mathrm{o}}}_{\mathrm{2}}}{\mathrm{C}}{{{\mathrm{O}}}_{\mathrm{2}}}}}{\mathrm{\ - \ }}{{E}_{{\mathrm{R}}{{{\mathrm{u}}}_{\mathrm{3}}}{\mathrm{@M}}{{{\mathrm{o}}}_{\mathrm{2}}}{\mathrm{C}}{{{\mathrm{O}}}_{\mathrm{2}}}}}{\mathrm{\ - \ }}{{E}_{\mathrm{X}}}
\end{eqnarray*}

where ${{E}_{{\mathrm{X \ldots R}}{{{\mathrm{u}}}_{\mathrm{3}}}{\mathrm{@M}}{{{\mathrm{o}}}_{\mathrm{2}}}{\mathrm{C}}{{{\mathrm{O}}}_{\mathrm{2}}}}}$, ${{E}_{{\mathrm{R}}{{{\mathrm{u}}}_{\mathrm{3}}}{\mathrm{@M}}{{{\mathrm{o}}}_{\mathrm{2}}}{\mathrm{C}}{{{\mathrm{O}}}_{\mathrm{2}}}}}$ and ${{E}_{\mathrm{X}}}$ represent the total energies of the X···Ru_3_@Mo_2_CO_2_ adsorption system, Ru_3_@Mo_2_CO_2_ and free X species, respectively. In addition, the electron density difference of the Ru_3_@Mo_2_CO_2_ or X···Ru_3_@Mo_2_CO_2_ adsorption system was calculated by equation (2)


(2) \begin{eqnarray*}
\Delta \rho \ = {{\rho }_{{\mathrm{AB}}}} - {{\rho }_{\mathrm{A}}} - {{\rho }_{\mathrm{B}}}
\end{eqnarray*}

where ${{\rho }_{{\mathrm{AB}}}}$ is the electron density of the entire Ru_3_@Mo_2_CO_2_ (or X…Ru_3_@Mo_2_CO_2_ system), ${{\rho }_{\mathrm{A}}}$ is the electron density of the Ru_3_ cluster (or X), and ${{\rho }_{\mathrm{B}}}{\mathrm{\ }}$is the electron density of Mo_2_CO_2_ (or Ru_3_@Mo_2_CO_2_), respectively.

## RESULTS AND DISCUSSION

### Stability of Ru_3_ cluster on Mo_2_CO_2_

**Figure 1.**
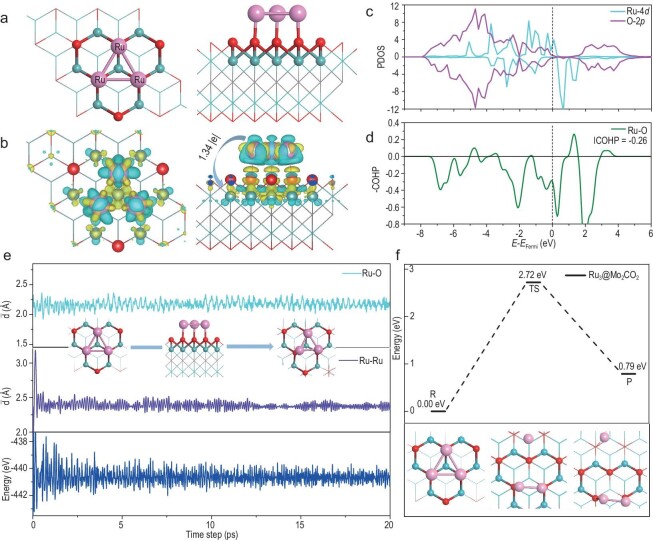
The stability and electronic properties of Ru_3_@Mo_2_CO_2_. (a) Optimized geometry and (b) calculated electron density difference of Ru_3_@Mo_2_CO_2_. (c) PDOS and (d) −COHP of Ru–O in Ru_3_@Mo_2_CO_2_. (e) Fluctuations of the total energy and average bond lengths of Ru–Ru in the Ru_3_ cluster and Ru–O on the Ru_3_@Mo_2_CO_2_ during AIMD simulations at a temperature of 473 K. (f) The dissociation of Ru_3_ to Ru_2_ and Ru_1_.

The optimized lattice parameters *a* = *b* = 2.86 Å for the primitive cell of Mo_2_CO_2_ are in agreement with the previous results (2.88 Å) [81]. Herein, the 3 × 3 supercell of Mo_2_CO_2_ support is used to anchor the Ru_3_ cluster, as shown in Fig. S1 of the supplementary data. The optimized stable structure of Ru_3_@Mo_2_CO_2_ is displayed in Fig. 1a, in which three Ru–O bonds were obviously formed after anchoring the Ru_3_ cluster onto Mo_2_CO_2_ of Ru_3_@Mo_2_CO_2_, with the three Ru–O bond lengths all at 2.14 Å, which is close to the Ru–O single bond distance [82]. The electron density difference and Bader charge analysis indicate that electron densities are enriched between Ru_3_ and the three surface O atoms of Mo_2_CO_2_, and the charge of 1.34 |e| is transferred from the Ru_3_ cluster to Mo_2_CO_2_, as shown in Fig. 1b. Furthermore, the calculated partial density-of-states (PDOS) of Ru_3_@Mo_2_CO_2_ (Fig. 1c) shows that there is a relatively large overlap between Ru 4*d* orbitals of the Ru_3_ cluster and O 2*p* orbitals of the three surface O atoms at the occupying states near the Fermi level, and the integrated crystal orbital Hamiltonian population (−ICOHP) value (−0.26) of Ru–O in Ru_3_@Mo_2_CO_2_ (Fig. 1d) demonstrates Ru and O interaction states lie in the bonding region below the Fermi level, indicating that the Ru_3_ cluster is strongly anchored on Mo_2_CO_2_ MXene for forming a Ru_3_@Mo_2_CO_2_ SCC. The Ru_3_-MXene interaction is therefore a result of the Ru–O covalent bonding, and the metal cluster–support interaction dictates the charge transfer, the oxidation state of Ru and the catalytic properties of Ru_3_@Mo_2_CO_2_ for N_2_-to-NH_3_ conversion.

To further explore the stability of Ru_3_ clusters on Mo_2_CO_2_ in a Ru_3_@Mo_2_CO_2_ SCC, AIMD simulations were performed at 473 and 673 K for 20 ps, as shown in Fig. 1e and Fig. S2, respectively. Starting from the optimized stable structure of the Ru_3_@Mo_2_CO_2_ SCC, the geometric configuration of the anchored Ru_3_ cluster on the surface of Mo_2_CO_2_ largely maintains a regular triangular structure, and the statistical average bond lengths of Ru–Ru ($\bar{d}$_Ru–Ru_) and Ru–O ($\bar{d}$_Ru–O_) are 2.39 and 2.17 Å, respectively, showing that the Ru_3_ cluster binds strongly on the surface of Mo_2_CO_2_. Moreover, with regard to the stability of the Ru_3_ cluster, Fig. 1f shows that the dissociation of one Ru atom from the Ru_3_ cluster in Ru_3_@Mo_2_CO_2_ reactant (R), yielding the metastable Ru single atom and Ru_2_ dimer as products (P), requires a high energy barrier of 2.72 eV (TS) and an endothermic reaction energy of 0.79 eV. These results demonstrate the stability of Ru_3_ clusters on Mo_2_CO_2_ support in Ru_3_@Mo_2_CO_2_ SCCs.

### Adsorption of N_2_ and H_2_

**Figure 2.**
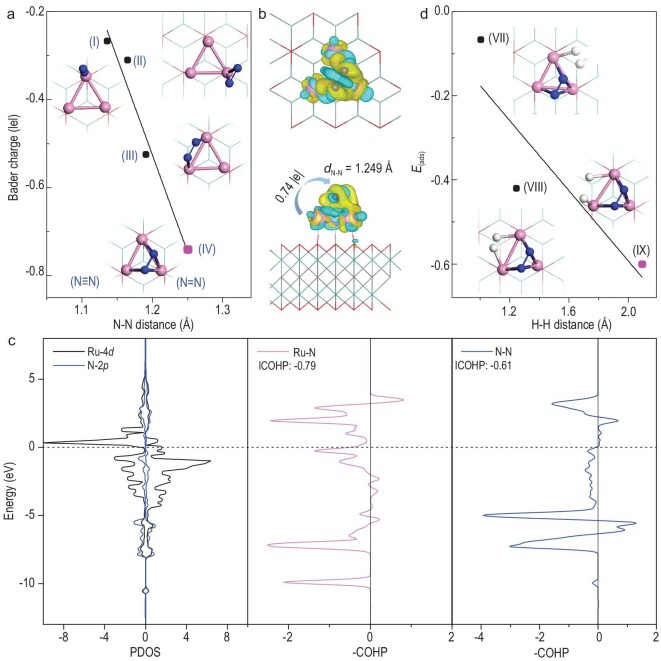
The stability and electronic properties of N_2_/H_2_ adsorbed on Ru_3_@Mo_2_CO_2_. (a) The correlation of the N−N bond lengths and Bader charges of *N_2_. (b) Electron density difference of *N_2_ and Ru_3_@Mo_2_CO_2_. (c) PDOS and −COHP of Ru−N. (d) The correlation of the H–H distance and the adsorption energies of *H_2_ with the co-adsorption of N_2_ + H_2_ on Ru_3_@Mo_2_CO_2_.

It is well known that the degree of N_2_ activation plays a crucial role in thermal N_2_-to-NH_3_ conversion, especially for the first associative reaction step of *N_2_ + *H → *NNH, which is often the rate-determining step (RDS) for NH_3_ synthesis [37,83]. Here we firstly investigate the possible configurations of N_2_ adsorption on the surface of Ru_3_@Mo_2_CO_2_ (Fig. 2a). Due to the strong N≡N triple bond, the N_2_ prefers molecular adsorption in the end-on and side-on modes rather than dissociative adsorption on the positively charged Ru_3_ cluster of Ru_3_@Mo_2_CO_2_. As shown in Fig. 2a and Table S1, the most stable adsorption mode of N_2_ is the end-on adsorption (I) at one Ru atom of the Ru_3_ cluster with an adsorption energy of −1.37 eV, in which the bond length of *N_2_ is 1.16 Å and the Bader charge of *N_2_ is only −0.26 |e|, suggesting that the adsorbed N_2_ in the end-on adsorption mode is weakly activated. However, the metastable N_2_ adsorbed in side-on adsorption mode can be well activated by three Ru atoms of the Ru_3_ cluster in IV (rather than by one or two Ru atoms in II and III), as shown in Fig. 2a. This strong adsorption arises from a significant charge transfer of 0.74 |e| from Ru_3_@Mo_2_CO_2_ to the adsorbed N_2_ with an N–N bond length of 1.25 Å in IV, which is close to the N–N double bond length of 1.20 Å [82]. In comparison, there is only 0.34 |e| charge transfer from isolated Ru_3_ clusters to the adsorbed N_2_ with an N–N bond length of 1.20 Å, indicating that the multiple-atom site of the SCC is advantageous for N_2_ activation [84,85].

Consistent with previous studies [86–88], the two adsorption modes of N_2_ can be transformed into each other at room temperature. Here, N_2_ on the Ru_3_ cluster of Ru_3_@Mo_2_CO_2_ is easily converted from end-on adsorption (I) to side-on adsorption (IV), with an energy barrier of only 0.55 eV, as shown in Fig. S3. Moreover, the calculated charge density difference (Fig. 2b) of structure IV shows that N_2_ is activated by Ru_3_@Mo_2_CO_2_ with strong Ru–N bonds formed, leading to a decrease in charge density on N–N and the enrichment of charge density on Ru–N. Furthermore, PDOS and −ICOHP electronic properties analysis of structure IV shows that the (N_2_)3*σ*→(Ru)4*d* donation and (Ru)4*d*→(N_2_)1π* back-donation interactions result in the side-on adsorbed N_2_ on Ru_3_@Mo_2_CO_2_, which further facilitates N_2_ activation, as shown in Fig. 2c.

The dissociative adsorption of H_2_ on the surface of Ru_3_@Mo_2_CO_2_ may also play an important role in the catalytic processes of NH_3_ synthesis, as shown in a previous study [89]. The adsorption of H_2_ and co-adsorption of H_2_ + N_2_ on Ru_3_@Mo_2_CO_2_ are explored. As shown in Fig. 2d, Fig. S4a and Table S2, H_2_ can easily undergo dissociative adsorption on the Ru_3_ cluster of Ru_3_@Mo_2_CO_2_ and the calculated dissociative adsorption energies of H_2_ in structures Ⅴ and Ⅵ are −1.01 and −1.03 eV, respectively, which are less than that (−1.31 eV) of N_2_ adsorption on the Ru_3_ cluster of Ru_3_@Mo_2_CO_2_ in structure IV, suggesting that N_2_ is preferentially adsorbed on the Ru_3_ cluster. Therefore, based on structure IV, the dissociatively adsorbed H_2_ can co-adsorb with the adsorbed N_2_ on the Ru_3_ cluster of Ru_3_@Mo_2_CO_2_, as shown in Fig. 2d. The most stable co-adsorption configuration of H_2_ + N_2_ is structure IX, for which the distance (2.09 Å) of H and H atoms is longer than that in structures VII and VIII. Moreover, it is evident that a Ru–H bond is formed in structure IX due to the obvious electron density accumulation between the two adsorbed H atoms and the Ru_3_ cluster (Fig. S4b). The calculated PDOS and −ICOHP of structure IX, where Ru–H interacting states lie in the bonding region below the Fermi level (Fig. S4c), indicate that H_2_ is also strongly dissociatively adsorbed on Ru_3_@Mo_2_CO_2_.

### Thermal N_2_-to-NH_3_ conversion mechanisms

**Figure 3.**
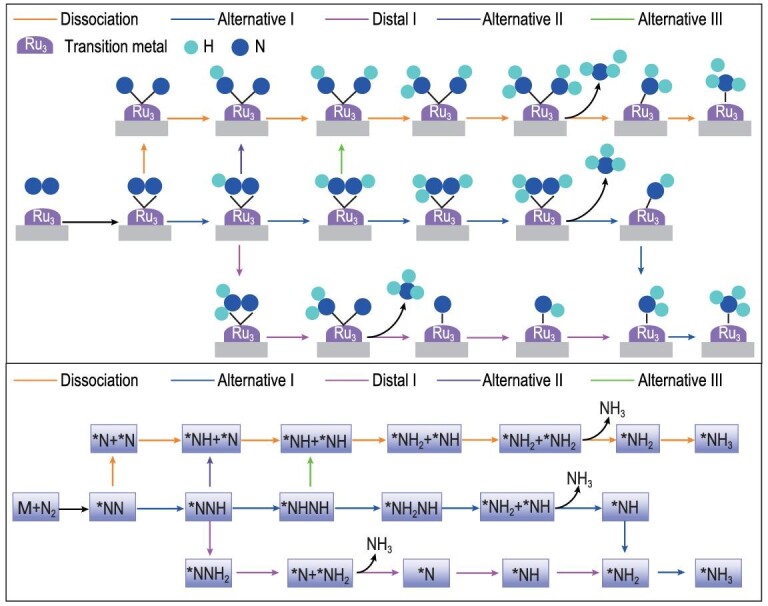
Schematic diagram of the possible reaction pathways of N_2_ to NH_3_ on Ru_3_@Mo_2_CO_2_ (* denotes surface-adsorbed species).

To study the thermal conversion of N_2_ to NH_3_ catalyzed by Ru_3_@Mo_2_CO_2_, the possible associative and dissociative reaction mechanisms are explored. The schematic diagram in Fig. 3 shows four possible associative and one dissociative reaction pathway for N_2_-to-NH_3_ conversion. For the associative mechanism, after the first hydrogenation of the activated N_2_ to form *NNH, it is either further hydrogenated to form *NHNH (alternative I) or *NNH_2_ (distal I), or dissociated into *N + *NH (alternative II), respectively. Moreover, *NHNH can further decompose into *NH + *NH in the alternative Ⅲ pathway. However, for the dissociative mechanism, the activated N_2_ is firstly dissociated into two *N on Ru_3_ cluster, and then the two *N are gradually hydrogenated to form NH_3_. Detailed schematic descriptions of the five reaction pathways for the thermal conversion of N_2_ to NH_3_ catalyzed by Ru_3_@Mo_2_CO_2_, and the calculated energy profiles for these reaction pathways with the corresponding optimized structures, are shown in Fig. 3 and Figs S5–S7, respectively.

**Figure 4.**
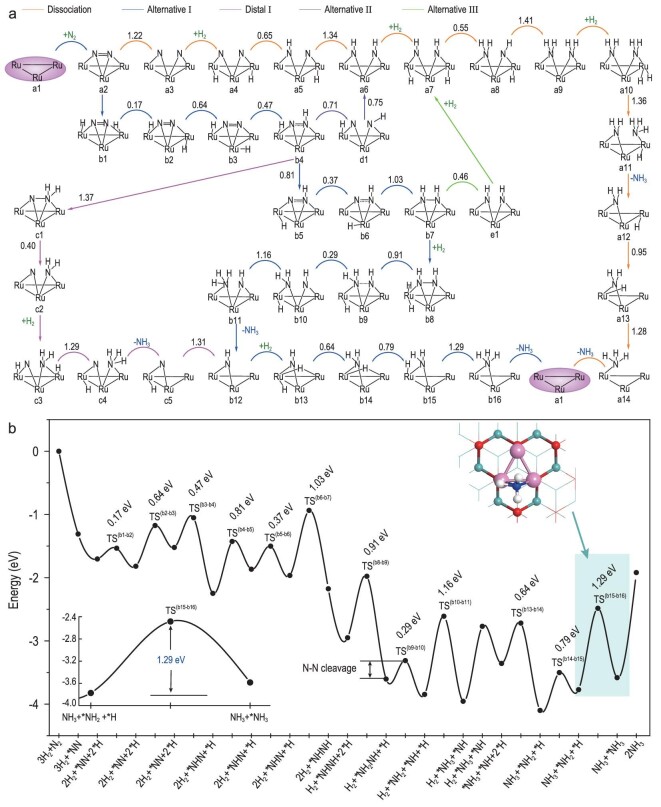
(a) Predicted possible reaction pathways of NH_3_ synthesis on Ru_3_@Mo_2_CO_2_. (b) Energy profile of the optimal associative alternative pathway I.

Because of the strong triple bond of N_2_, for most iron and ruthenium metal-based catalysts [11,23,28,90–93], the dissociative step of *N_2_ → *N + *N is often identified as the RDS for NH_3_ thermal synthesis. On the B5 site of the bulk Ru metal surface, the N_2_ dissociation barrier is expected to be much lower than on supported clusters because of the Ru(0) oxidation state. However, as shown in Fig. 4a and Fig. S5, for Ru_3_@Mo_2_CO_2_ the N–N bond cleavage barrier energy in the dissociative pathway is only 1.22 eV for the transition state of TS^(a2–a3)^ (a2 to a3), because of the synergistic effect [22,23] on the multiple-Ru sites of the Ru_3_ cluster in favor of N_2_ activation with an N–N bond length of 1.25 Å. With the hydrogenation of *N on the Ru_3_ cluster of Ru_3_@Mo_2_CO_2_, 1.34 eV barrier energy is required for TS^(a5–a6)^ to form *NH + *NH. Then a higher barrier energy of 1.41 eV (TS^(a8–a9)^) is needed to form *NH_2_ + *NH_2_ species in a9. Next, the two generated *NH_2_ are successively hydrogenated to form two NH_3_ from a10 to a11 with a barrier of 1.36 eV, and from a13 to a14 with a barrier of 1.28 eV. Therefore, it is obvious that the step from a8 to a9 with barrier energy of 1.41 eV is the RDS in the dissociative pathway of N_2_-to-NH_3_ conversion on Ru_3_@Mo_2_CO_2_ in Fig. 4a.

Compared to the direct breaking of the N–N bond of *N_2_ in the aforementioned dissociative mechanism, it is more favorable for *N_2_ hydrogenation to form *NNH in b4 with a barrier energy of 0.47 eV (TS^(b3–b4)^) through the associative pathways [22,23], and the bond length of N–N is stretched to 1.35 Å in b4, which is close to the N–N single bond [82]. In the alternative pathway I, the generated *NNH can be further hydrogenated into *HNNH through TS^(b6–b7)^ with a slightly high barrier energy of 1.03 eV, and the N–N bond length is further lengthened to 1.39 Å. Subsequently, the second H_2_ is dissociatively adsorbed on the Ru_3_ cluster in b8, and then one of the *H atoms attracts *HNNH species to form *HNNH_2_ with a barrier of 0.91 eV (TS^(b8–b9)^), followed by the N–N bond breaking to form *NH and *NH_2_ (0.29 eV, TS^(b9–b10)^). Then the remaining *H attacks *NH_2_ to yield the first NH_3_, with a barrier energy of 1.16 eV from b10 to b11. Finally, the remaining *NH is hydrogenated twice to yield the second NH_3_ from b15 to b16, in which the step of *NH_2_ + *H → *NH_3_ is the RDS in the alternative pathway I for NH_3_ synthesis, with a high barrier energy of 1.29 eV, as shown in Fig. 4a and b, and Fig. S6.

Associative distal pathway I is different from alternative pathway I starting from b4, where the *NNH species is further hydrogenated into *NNH_2_ with a slightly high barrier of 1.37 eV (TS^(b4–c1)^), and the N≡N triple bond is approximately activated to single bond (1.41 Å) in *NNH_2_ species by the Ru_3_ cluster. Thus, the NNH_2_ is easily broken into *N and *NH_2_ on the Ru_3_ cluster (0.40 eV, TS^(c1–c2)^). Furthermore, the subsequent hydrogenation reactions of *NH_2_ + *H → NH_3_ (c3 to c4) and *N + *H → *NH (c5 to b12) are more easily compared to the formation of *NNH_2_, owing to the two lower barrier energies of 1.29 and 1.31 eV, as shown in Fig. 4a and Fig. S7. Accordingly, the formation of *NNH_2_ is the RDS in associative distal pathway I for the conversion of N_2_ to NH_3_.

For the associative alternative pathways in Fig. 4a and Fig. S7, based on the structures of b4 and b7, the N–N bonds in *NNH and *HNNH species are easy to break directly in alternative pathway II from b4 to d1 (0.71 eV, TS^(b4–d1)^) and in alternative pathway III from b7 to a6 (0.46 eV, TS^(b7–e1)^), respectively. Then, the resulting *N and *NH species undergo hydrogenation along the dissociation pathway to form NH_3_.

Overall, the N–N bond cleavage energy barrier (0.71 eV, TS^(b4–b5)^) in *NNH is obviously lower than that (1.22 eV, TS^(a2–a3)^) in *N_2_, resulting in NH_3_ synthesis preferentially following the associative pathway on Ru_3_@Mo_2_CO_2_ under moderate thermodynamic conditions. The RDSs of the above five reaction pathways all involve the hydrogenation steps for the formation of *NH*_x_* species, and the reaction barrier energies are 1.29 eV (b15 to b16) in alternative pathway I, 1.37 eV (b4 to c1) in distal pathway I and 1.41 eV (a8 to a9) in alternative pathway II, Ⅲ and dissociation mechanisms (Fig. 4a and Fig. S5), respectively, showing that efficient thermal N_2_-to-NH_3_ conversion on the Ru_3_ cluster supported by Mo_2_CO_2_ MXene is dominated by associative alternative pathway I at mild conditions.

To further explore the effect of thermodynamic conditions, the Gibbs free energy profile is calculated at 48 bar and 700 K. As shown in Fig. S8, although the free energy changes slightly, the optimal reaction pathway and the RDS remain unchanged, where the energy barrier only increases by 0.02 eV.

### Origin of N_2_-to-NH_3_ conversion on Ru_3_@Mo_2_CO_2_

**Figure 5.**
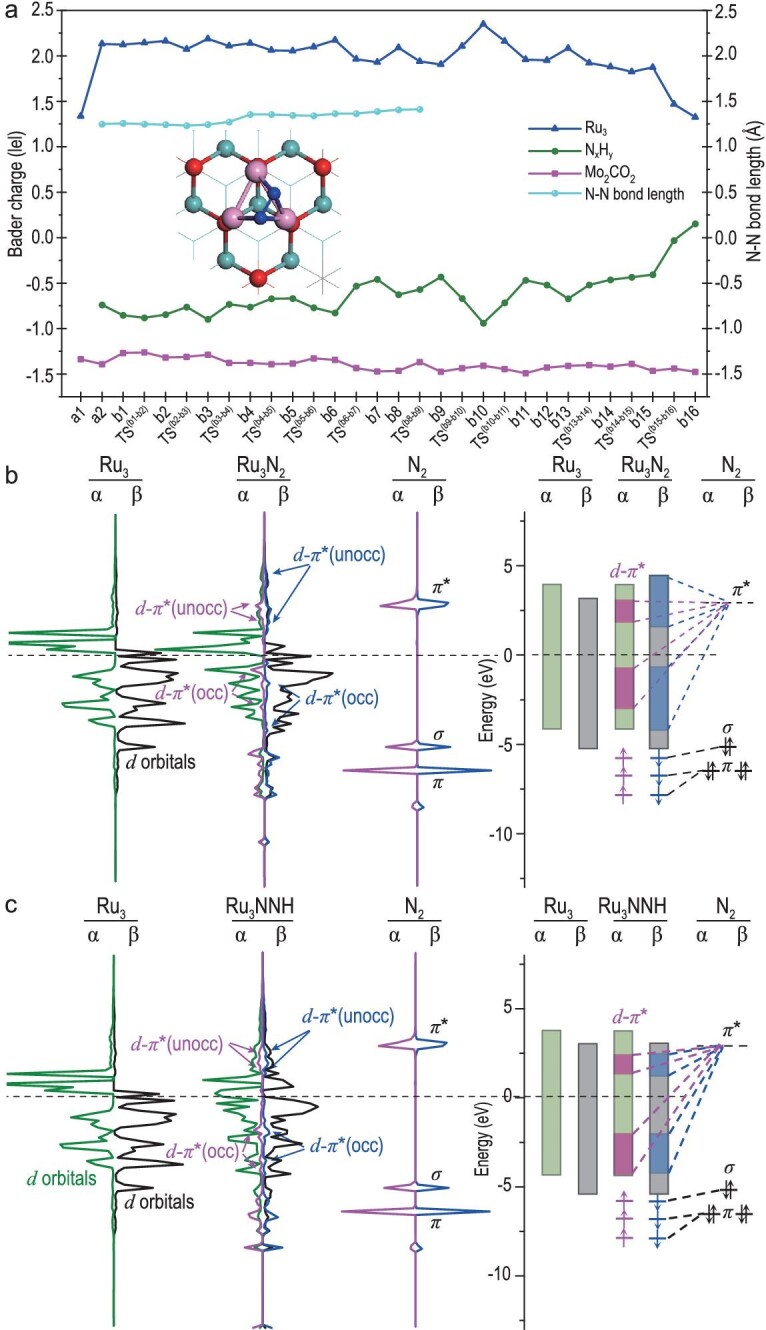
(a) The Bader charge variation of the adsorbates of the Ru_3_ cluster, N*_x_*H*_y_* species, dinitrogen and Mo_2_CO_2_ support, and the changes of N–N bond lengths at every step of the catalytic cycle along the optimal associative alternative pathway I. (b) PDOS and schematic illustrations of the 4*d* orbitals of the Ru_3_ cluster on Mo_2_CO_2_, 2*p*-orbitals of the N_2_ gas molecule, and their interaction within Ru_3_N_2_/Mo_2_CO_2_. (c) PDOS and schematic illustrations for Ru_3_NNH/Mo_2_CO_2_.

The configurations of the active center, support and the intermediate species, especially the oxidation states of the active center, are the key [94,95] to understanding the essence of NH_3_ synthesis through the multi-step redox reactions catalyzed by Ru_3_@Mo_2_CO_2_. Therefore, we further analyzed the Bader charges of Mo_2_CO_2_ support, Ru_3_ cluster, N*_x_*H*_y_* species and N_2_, and the changes of N–N bond length along the optimal associative alternative pathway I for the N_2_-to-NH_3_ conversion, as shown in Fig. 5 and Tables S3 and S4. As seen from Fig. 5 and Table S3, the Bader charge of Mo_2_CO_2_ support is about −1.40 |e| and remains almost unchanged throughout the whole process of NH_3_ synthesis, indicating that Mo_2_CO_2_ support here serves as the electron reservoir for the anchored Ru_3_ cluster, which tends to effectively adsorb and activate N_2_. This originates from its ability to effectively accept the electron lone pair of N_2_, thereby decreasing the bonding of N_2_. Furthermore, the oxidation state of the anchored Ru_3_ cluster significantly increases after the adsorption of N_2_, with its Bader charge increasing from +1.34 |e| in a1 to +2.13 |e| in a2. This charge transfer arises from the back-donation of Ru *α*-spin *d*-electrons to the antibonding orbital of N_2_ through *d*-π* orbital interaction, which involves the energy-level-matched d orbitals of the Ru_3_ cluster and the antibonding π* orbital of N_2_, as shown in Fig. 5. Thus, the N–N bond is significantly weakened so that the hydrogenation of the activated N_2_ becomes easier than that on the isolated Ru_3_ cluster [92].

After the first hydrogenation of N_2_, the Ru_3_ cluster further back-donates its *β*-spin *d*-electrons to the antibonding orbital of HNN, resulting in its lower hydrogenation (0.47 eV) and dissociation energy barriers (0.71 eV) than those of N_2_. Moreover, the change of the Bader charges of N*_x_*H*_y_* species and the Ru_3_ cluster are complementary in the whole of associative alternative pathway I, indicating that Ru_3_ serves as a reservoir to regulate the charge transfer for N_2_ synthesis, in which the calculated Bader charges of Ru_3_ range from +1.82 to +2.13 |e|. After the *NH_3_ desorbs from the surface of Ru_3_@Mo_2_CO_2_, the oxidation state of the Ru_3_ cluster returns to its initial low valence state from b15 to b16. It appears that the change of the Bader charges of N*_x_*H*_y_* species and the Ru_3_ cluster are complementary in the whole process of NH_3_ synthesis (Fig. 5a), showing that charges transfer from the anchored Ru_3_ cluster to the adsorbed N_2_ and N*_x_*H*_y_* species to further facilitate N_2_ hydrogenation during the process of N_2_-to-NH_3_ conversion.

In addition, the complete cleavage of the N≡N triple bond undergoes five stepwise stages in optimal associative alternative pathway I (Fig. 5a and Table S4): N_2_ (g) $\mathop \to \limits^{\bigcirc{1}} $ *N_2_  $\mathop \to \limits^{\bigcirc{2}} $ *NNH $\mathop \to \limits^{\bigcirc{3}} $ *NHNH $\mathop \to \limits^{\bigcirc{4}} $ *NH····NH_2_  $\mathop \to \limits^{\bigcirc{5}} $ *NH_2_ + *NH. The bond length of N–N changes from 1.08 Å in gas-phase N_2_ to an N–N double bond of 1.25 Å in *N_2_, to an approximate N–N single bond of 1.35 Å in *NNH, to an N–N single bond of 1.41 Å in *NHNH, to an N–N distance of 1.48 Å with very weak interaction in *NH and *NH_2_, until the N–N bond complete cleavage (3.31 Å) for *NH_2_ and *NH.

### Microkinetic simulations of N_2_-to-NH_3_ conversion on Ru_3_@Mo_2_CO_2_

**Figure 6.**
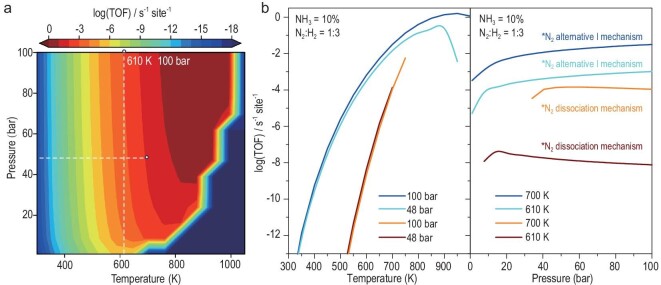
Microkinetic simulations. (a) TOF per site of ammonia synthesis on Ru_3_@Mo_2_CO_2_ mapped with pressure (1–100 bar) and temperature (300–1050 K). H_2_: N_2_ = 3 : 1, and the conversion ratio of NH_3_ is 10%. (b) TOF contributions from the alternative pathway-I mechanism and the dissociative mechanism at constant pressures of 100 and 48 bar and constant temperatures of 700 and 610 K, respectively.

To directly examine the catalytic activity of N_2_-to-NH_3_ conversion under realistic conditions, we further performed microkinetic simulations to estimate the TOF. The TOF is calculated under a pressure range of 1–100 bar and a temperature range of 300–1050 K. As shown in Fig. 6 and Tables S5 and S6, the TOF of the N_2_-to-NH_3_ conversion on the Ru_3_@Mo_2_CO_2_ SCC is <10^−10^ s^−1^ site^−1^ below 400 K, due to its RDS energy barrier of 1.29 eV. With an increase in temperature, the TOF rapidly increases. However, at a pressure of 1 bar, when the temperature exceeds 640 K, the TOF decreases with the increase in temperature, which is due to the influence of the entropy effect and the fact that N_2_ cannot stably adsorb on the metal for reaction. With the increased pressure, the TOF continues to increase. When the temperature reaches 610 K and the pressure reaches 100 bar, the reaction easily overcomes the energy barrier of the RDS and the TOF of N_2_-to-NH_3_ conversion of Ru_3_@Mo_2_CO_2_ is 1.02 × 10^−3^ s^−1^ site^−1^. Moreover, the catalytic performance of Ru_3_@Mo_2_CO_2_ for ammonia synthesis even surpasses that of the well-known Ru B5 site on Ru-metal and Fe_3_/θ-Al_2_O_3_(010) [23,96] due to its TOF reaching 1.45 × 10^−2^ s^−1^ site^−1^ at 48 bar and 700 K. Furthermore, we further compare the TOF of the N_2_-to-NH_3_ conversion on Ru_3_@Mo_2_CO_2_ through the dissociation pathway and the association alternative pathway I. As shown in Fig. 6, in the range of the temperature and pressure considered, the TOF in alternative pathway I is significantly larger than that in the dissociation pathway, which further confirms that the ammonia synthesis on Ru_3_@Mo_2_CO_2_ will prefer alternative pathway I, which can bypass the restriction of BEP relation.

Moreover, we further explored the evolution of surface coverage at 48 bar and 700 K using MKMCXX code [77]. As shown in Fig. S9, at this thermodynamic condition, as the reaction progresses, the coverage degree of H and N_2_ first increases and then decreases, while the coverage degree of intermediate NH_2_* increases continuously, indicating that the synthesis of ammonia can proceed smoothly at 48 bar and 700 K conditions.

## CONCLUSIONS

The first-principles calculations have been performed on the stability and electron structure of Ru_3_@Mo_2_CO_2_, and its catalytic performance for ammonia synthesis from nitrogen has been investigated. It is shown that Ru_3_@Mo_2_CO_2_ has high stability, originating from the strong bonding interaction between the anchored Ru_3_ cluster and its surrounding O atoms. N_2_ is found to stably adsorb on Ru_3_@Mo_2_CO_2_ by both end-on and side-on adsorption modes, and gets obviously activated due to the *d*-*π* interaction between N_2_ and the Ru_3_ cluster.

Four possible mechanisms for ammonia synthesis, including one dissociation mechanism and three association mechanisms, are studied in detail. The results show that association alternative pathway I is the most feasible for N_2_-to-NH_3_ conversion on Ru_3_@Mo_2_CO_2_, in which the RDS is NH_2_* + H* → NH_3_* with a low energy barrier of 1.29 eV. The high catalytic performance of Ru_3_@Mo_2_CO_2_ for ammonia synthesis originates from the anchored Ru_3_ cluster, which can serve as an electron reservoir to effectively regulate the charge transfer during the ammonia synthesis process, with the assistance of the support Mo_2_CO_2_. Moreover, microkinetic analysis shows that the TOF of N_2_-to-NH_3_ conversion on Ru_3_@Mo_2_CO_2_ via association alternative pathway I is as high as 1.45 × 10^−2^ s^−1^ site^−1^ at 48 bar and 700 K. The theoretical results show that Ru_3_@Mo_2_CO_2_ is a promising SCC for ammonia synthesis directly from nitrogen and hydrogen. This work demonstrates the potential and capacity of SCCs featuring atomically precise active sites in the rational design of high-performance heterogeneous catalysts.

## Supplementary Material

nwae251_Supplemental_File
