# Supplementary material for: Ru3@Mo2CO2 MXene single-cluster catalyst for highly efficient N2-to-NH3 conversion
Source: Natl Sci Rev. 2024 Jul 26;11(9):nwae251. doi: 10.1093/nsr/nwae251 (PMC11385201; doi:10.1093/nsr/nwae251)
Supplement: nwae251_Supplemental_File [file nwae251_supplemental_file.pdf]

$Ru_3@Mo_2CO_2$ MXene Single-Cluster Catalyst for Highly Efficient N_2-to-NH_3 Conversion

Cong Zhang,[a] Ze-Hui Wang,[b,c] Haiyan Wang,[a] Jin-Xia Liang,*[,a] Chun Zhu*[,a,c] and Jun Li*[,c,d,e]

[a]School of Chemistry and Chemical Engineering, Guizhou University, Guiyang 550025, China

[b]Shaanxi Key Laboratory of Catalysis, Institute of Theoretical and Computational Chemistry, Shaanxi University of Technology, Hanzhong 723000, China

[c]Department of Chemistry and Guangdong Provincial Key Laboratory of Catalytic Chemistry, Southern University of Science and Technology, Shenzhen 518055, China

[d]Department of Chemistry and Engineering Research Center of Advanced Rare-Earth Materials of Ministry of Education, Tsinghua University, Beijing 100084, China

[e]Fundamental Science Center of Rare Earths, Ganjiang Innovation Academy, Chinese Academy of Sciences, Ganzhou 341000, China

*Corresponding authors. E-mail: liangjx2009@163.com; czhu2014@163.com; junli@tsinghua.edu.cn

1. Computational details

For the reaction rate constant and free energy correlation, we calculated the reaction rates of the rate-determining steps (b15 → b16) in the distal I reaction mechanism for N_2 on $Ru_3@Mo_2CO_2$. The Eyring-Polanyi equation based on the transition state theory (TST)[1] is used to calculate the reaction rate constant, which can be written as Eq. (1):

$$k^{TST} = \sigma \frac{k_B T}{h} (\frac{RT}{P_0})^{\Delta n} e^{-\Delta G^{0,\neq}/k_B T} \qquad (1)$$

where:

σ is the transmission coefficient;

k_B is Boltzmann's constant;

T is the absolute temperature;

h is the Planck's constant;

R is the gas constant;

P_0 is the standard atmospheric pressure;

And $\Delta G^{0,\neq}$ is the free energy of activation, where:

$$\Delta G^{0,\neq}(T) = \Delta G^0_{TS}(TS) - \Delta G^0_{Reactant}(T) \quad (2)$$

The rate-determining step in distal I is the formation of NH_3 species. To ensure the reaction rate constant, we firstly calculate the free energy of activation. The vibrational partition function is written as Eq. (3):

$$q_{vib} = \pi \prod_i \frac{1}{1-e^{-\frac{\varepsilon_i}{k_B T}}} \qquad (3)$$

Where, ε_i is the vibrational energies.

The internal energy is written as:

$$U_{vib} = \sum_i \frac{\varepsilon_i}{e^{\frac{\varepsilon_i}{k_B T}}-1} \qquad (4)$$

And the entropy, S, is calculated via:

$$S_{vib} = k_B \sum_i \frac{\varepsilon_i}{k_B T(e^{\frac{\varepsilon_i}{k_B T}}-1)} - \ln 1 - e^{-\frac{\varepsilon_i}{k_B T}} \qquad (5)$$

Since the zero-point vibrational energy (ZPVE) has already been considered in the reaction energy profiles, the thermal correction to ΔG(T) in the present work at 298.15 K and P = 1 atm is defined as Eq. (6):

$$G_{corr}(298.15k) = U_{vib}(298.15k) + 298.15 * S_{vib}(298.15k) \qquad (6)$$

Thus, the free energy of activation $\Delta G^{0,\neq}$ for the rate-determining step of pathway III is 1.29 eV calculated from VASPkit postprocessing tool[2]. Accordingly, the calculated reaction rate constant is 2.41×10^{-8}/(s mol L^{-1}).

References:

1. Fernández-Ramos, A.; Ellingson, B. A.; Meana-Pañeda, R.; Marques, J. M. C.; Truhlar, D. G., Symmetry numbers and chemical reaction rates. *Theor. Chem. Acc.* **2007,** *118* (4), 813-826.
2. Wang, V.; Xu, N.; Liu, J.-C.; Tang, G.; Geng, W.-T., VASPKIT: A user-friendly interface facilitating high-throughput computing and analysis using VASP code. *Comput. Phys. Commun.* **2021,** *267,* 108033.

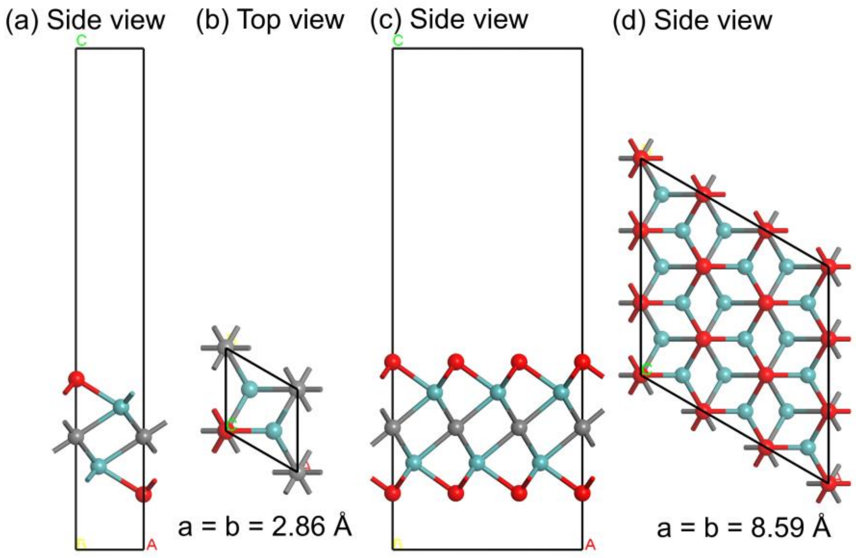

Figure S1. Optimized structures of the Mo_2CO_2 primitive cell and 3 × 3 supercell.

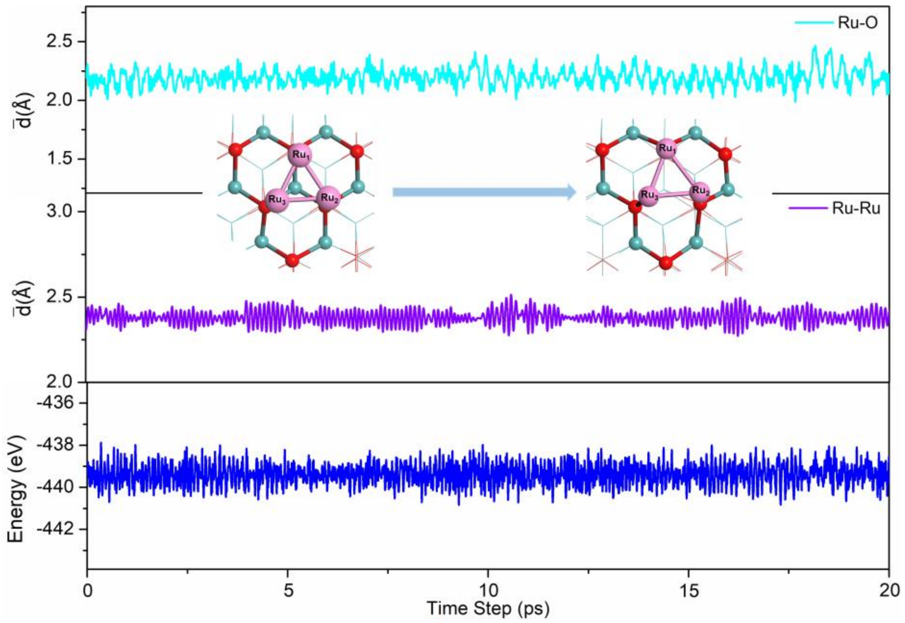

Figure S2. The total energy (eV), the average bond length (Å) of Ru-Ru in Ru_3 cluster and Ru-O fluctuation on the $Ru_3@Mo_2CO_2$ during AIMD simulations at 673 K.

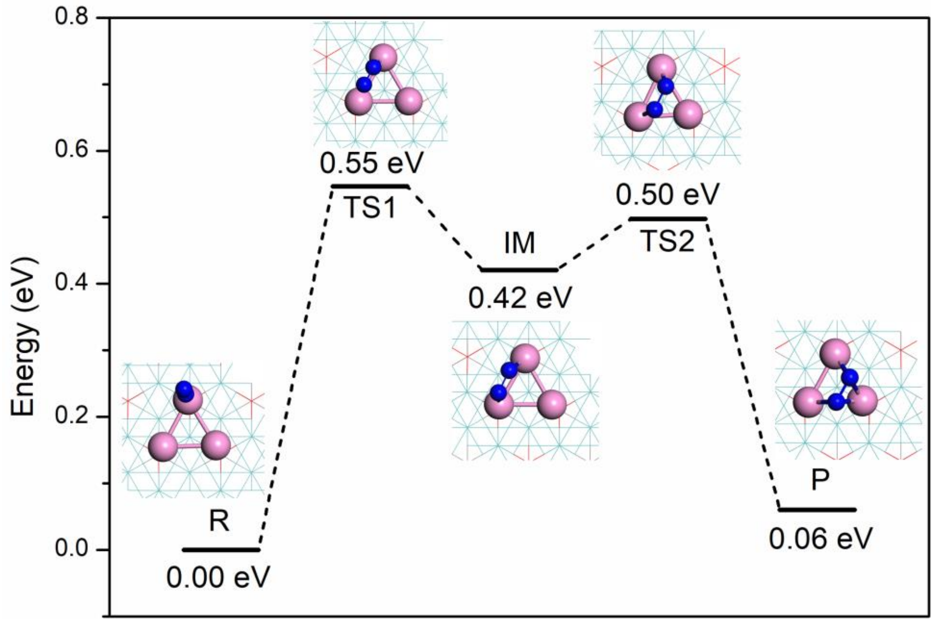

Figure S3. The predicted profile of reaction pathway for the transformation of dinitrogen configurations.

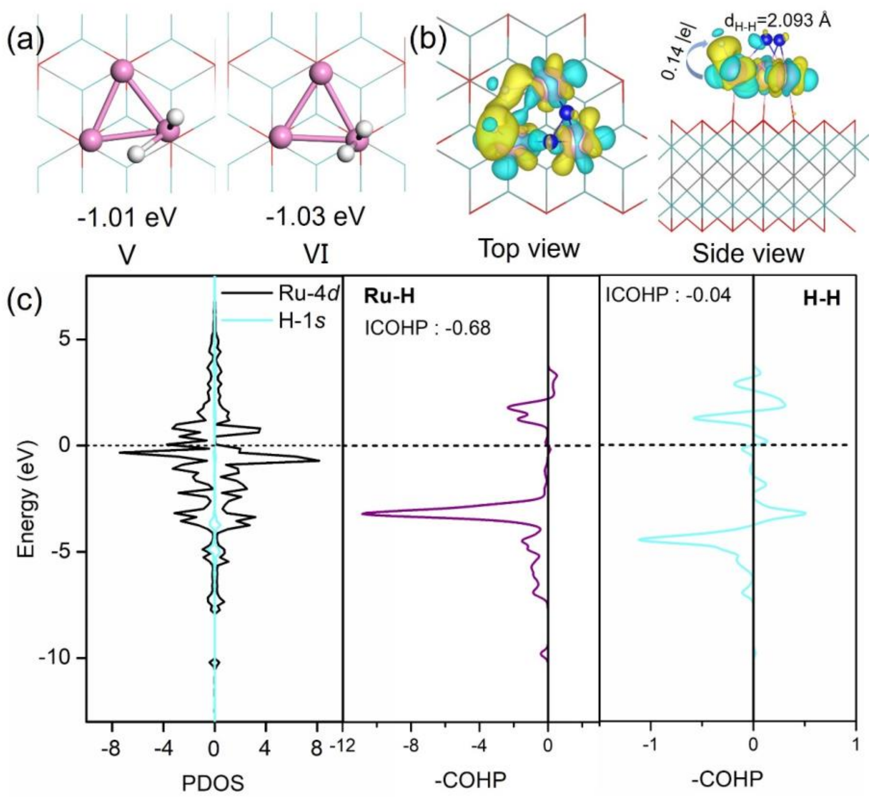

Figure S4. The stability and electronic properties of H_2 adsorbed on $Ru_3@Mo_2CO_2$. (a) The optimized geometry and (b) the calculated electron density difference of $Ru_3@Mo_2CO_2$ with H_2 and N_2 co-adsorption; (c) the PDOS and (d) the -COHP of Ru-H in $Ru_3@Mo_2CO_2$.

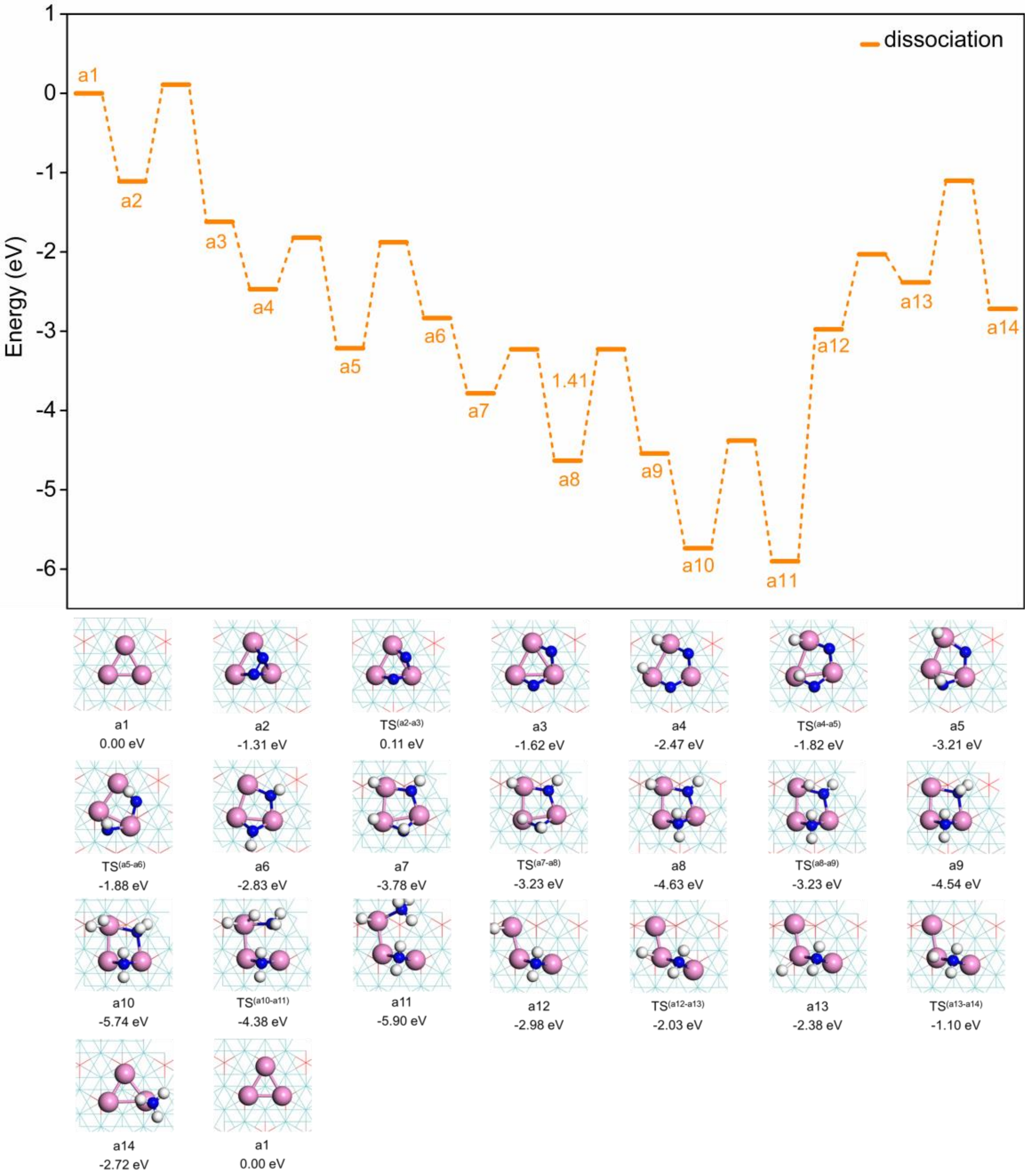

Figure S5. The predicted profile of dissociation pathway and the corresponding optimized structures for NH_3 synthesis.

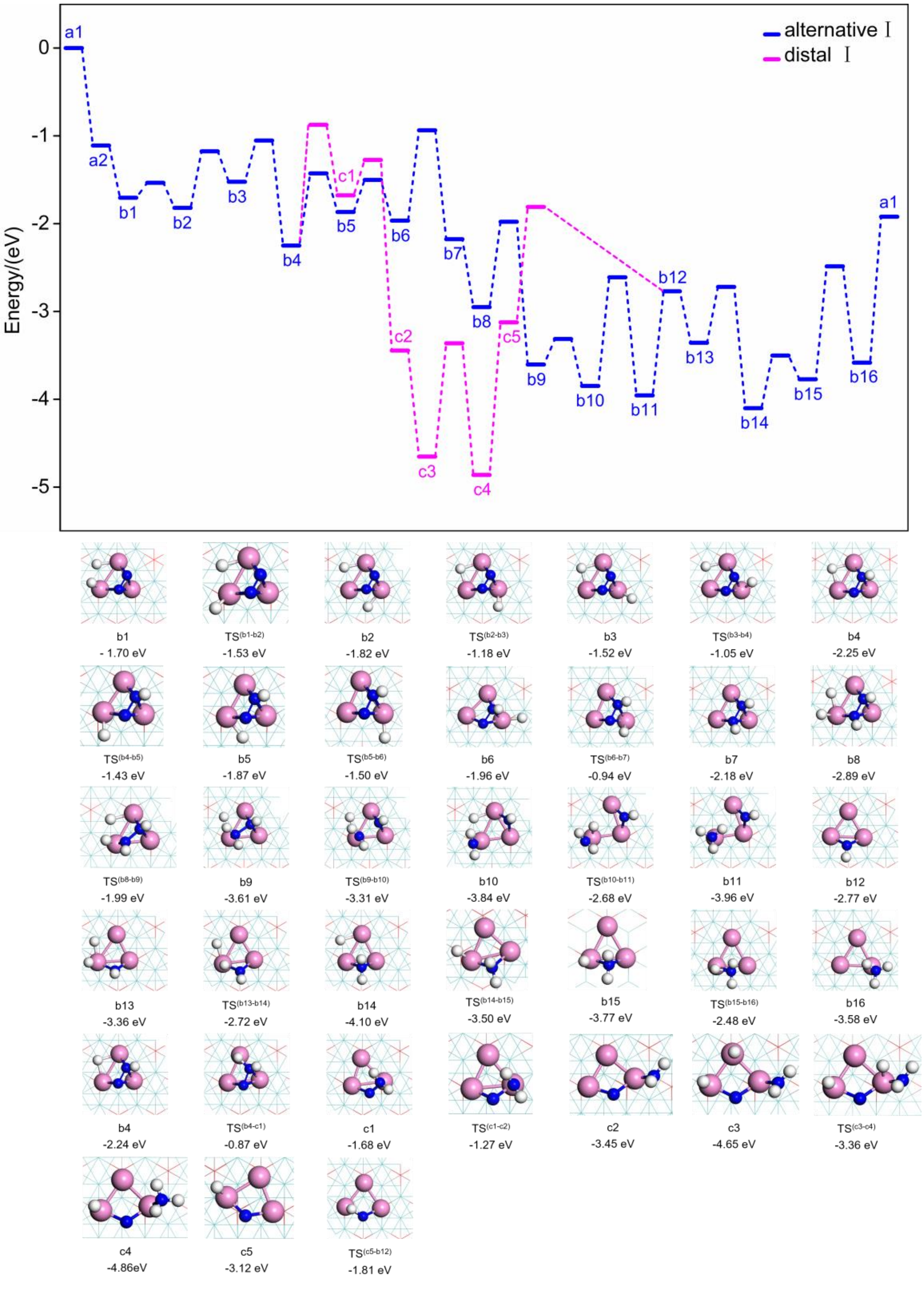

Figure S6. The predicted profile of the associative alternative pathway I and distal pathway I and the corresponding optimized structures for NH_3 synthesis.

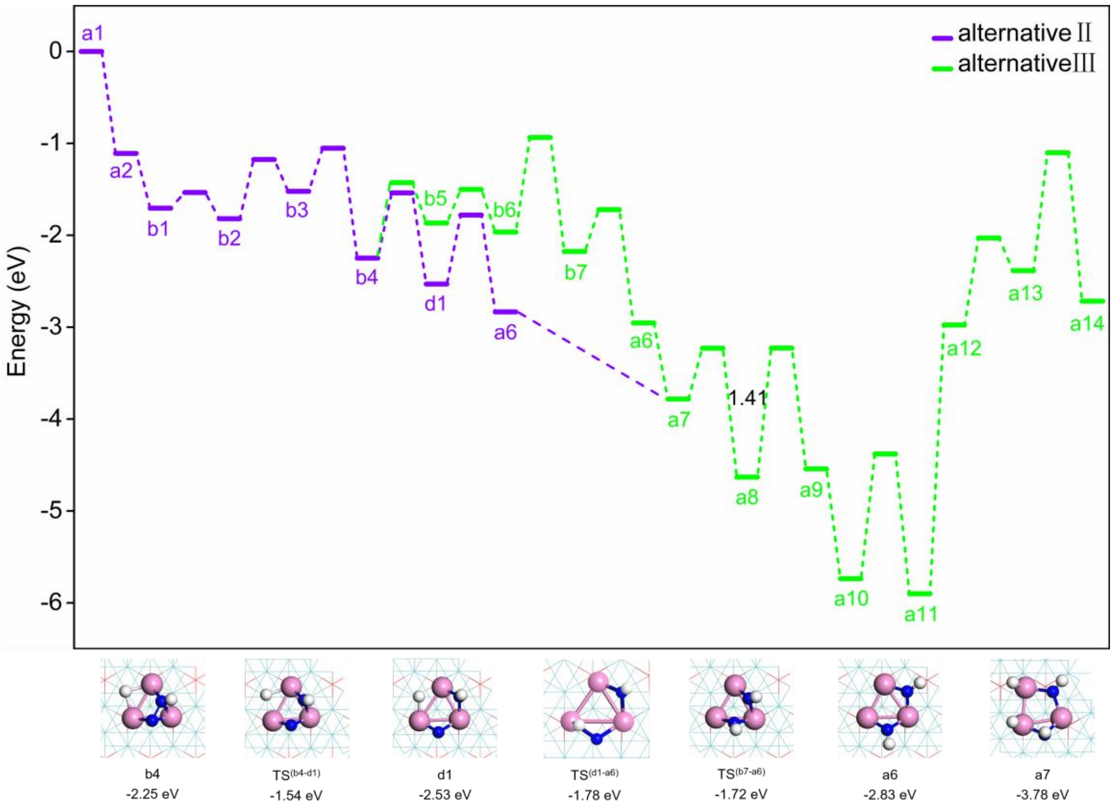

Figure S7. The predicted profile of the alternative pathways II and III and the corresponding optimized structures for NH_3 synthesis.

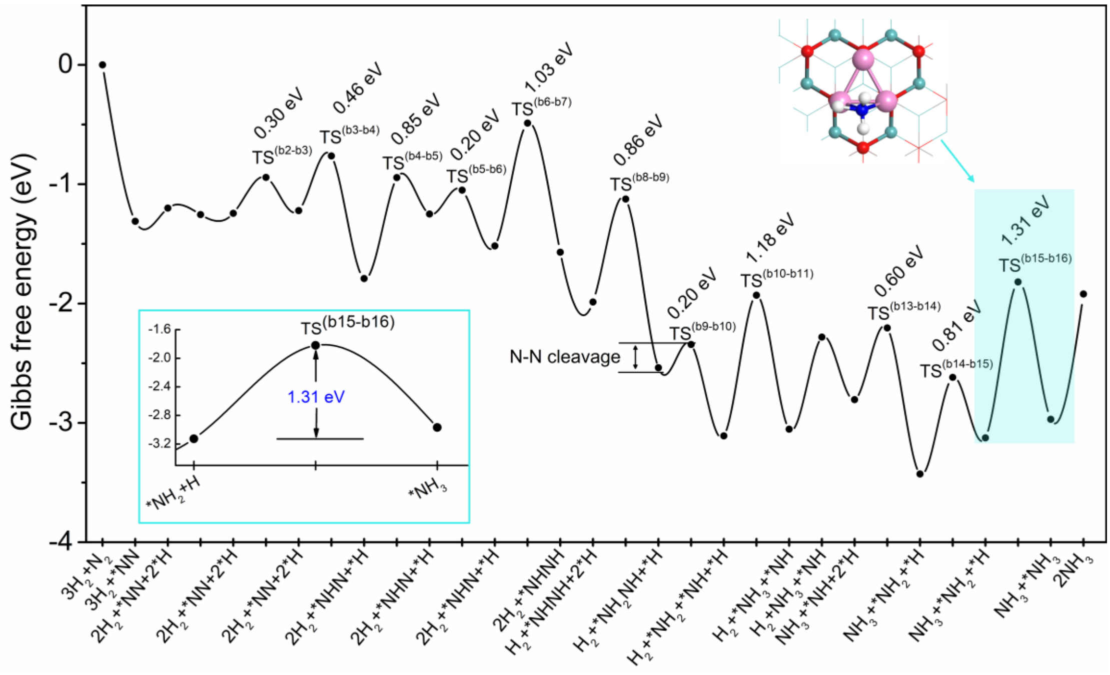

Figure S8. The energy profile of the optimal associative alternative pathway I at 700 K.

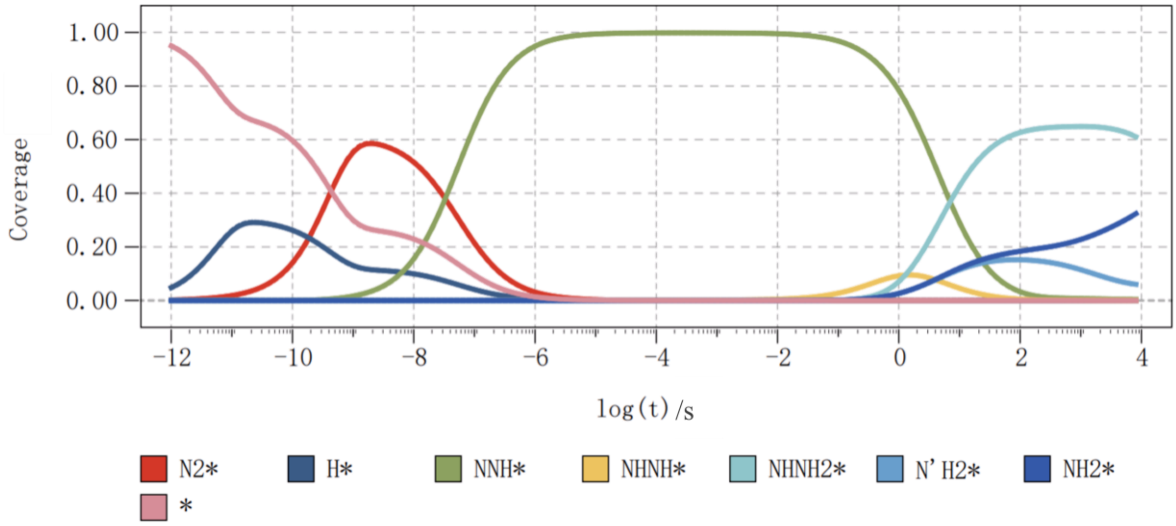

Figure S9. Coverage for surface species on $Ru_3@Mo_2CO_2$. Reaction conditions and parameters are: H_2:N_2 ratio = 3:1 and NH_3 conversion is fixed at 10%.

Table S1. The calculated adsorption energy (E_{ads}/eV), bond length (d_{N-N}/Å) and Bader charge (|e|) of N_2 adsorbed on $Ru_3@Mo_2CO_2$.

Entry	E_{ads}/eV	d_{N-N}/Å	Bader charge (|e|)
I	-1.37	1.14	-0.27
II	-0.69	1.16	-0.31
III	-1.11	1.19	-0.52
IV	-1.31	1.25	-0.74

Table S2. The calculated adsorption energy (E_{ads}/eV), bond length (d_{H-H}/Å) and Bader charge (|e|) of H_2 adsorbed on $Ru_3@Mo_2CO_2$.

Entry	E_{ads}	d_{H-H}/Å	Bader charge of N_2 (|e|)	Bader charge of H_2 (|e|)
V	-1.01	1.80	-	-0.26
VI	-1.03	0.99	-	-0.07
VII	-0.07	1.00	-0.62	-0.01
VIII	-0.42	1.24	-0.71	-0.01
IX	-0.60	2.09	-0.69	-0.14

Table S3. The N-N bond length change of dinitrogen during the NH_3 synthesis.

Entry	d_{N-N}/Å	Entry	d_{N-N}/Å
a2	1.25	b6	1.36
b1	1.26	$TS^{(b6-b7)}$	1.36
$TS^{(b1-b2)}$	1.25	b7	1.39
b2	1.24	b8	1.41
$TS^{(b2-b3)}$	1.23	$TS^{(b8-b9)}$	1.41
b3	1.24	b9	1.48
$TS^{(b3-b4)}$	1.27	$TS^{(b9-b10)}$	1.94
b4	1.35	b10	3.31
$TS^{(b4-b5)}$	1.36	$TS^{(b10-b11)}$	3.61
b5	1.35	b11	3.06
$TS^{(b5-b6)}$	1.34	-	-

Table S4. Calculated Bader charges (|e|) for Mo_2CO_2, N_xH_y, Ru_3, N_a and N_b in the structures of alternative pathway I.

Species	Mo_2CO_2	N_xH_y	Ru_3	N_a	N_b
a1	-1.34	-	1.34	-	-
a2	-1.39	-0.74	2.13	-0.34	-0.40
b1	-1.27	-0.85	2.12	-0.33	-0.36
$TS^{(b1-b2)}$	-1.26	-0.88	2.14	-0.30	-0.37
b2	-1.32	-0.85	2.16	-0.29	-0.37
$TS^{(b2-b3)}$	-1.31	-0.76	2.07	-0.28	-0.36
b3	-1.29	-0.90	2.19	-0.32	-0.35
$TS^{(b3-b4)}$	-1.38	-0.73	2.11	-0.34	-0.38
b4	-1.38	-0.76	2.14	-0.42	-0.69
$TS^{(b4-b5)}$	-1.39	-0.67	2.06	-0.41	-0.70
b5	-1.38	-0.67	2.05	-0.40	-0.72
$TS^{(b5-b6)}$	-1.33	-0.77	2.10	-0.41	-0.71
b6	-1.34	-0.83	2.17	-0.46	-0.71
$TS^{(b6-b7)}$	-1.43	-0.53	1.96	-0.43	-0.68
b7	-1.47	-0.46	1.93	-0.66	-0.72
b8	-1.46	-0.63	2.09	-0.70	-0.62
$TS^{(b8-b9)}$	-1.37	-0.57	1.94	-0.75	-0.67
b9	-1.47	-0.43	1.91	-0.77	-0.84
$TS^{(b9-b10)}$	-1.44	-0.67	2.11	-0.88	-1.00
b10	-1.41	-0.94	2.35	-0.99	-1.14
$TS^{(b10-b11)}$	-1.44	-0.72	2.16	-1.01	-1.12
b11	-1.49	-0.47	1.96	-1.02	-1.30
b12	-1.43	-0.52	1.95	-0.97	-
b13	-1.41	-0.67	2.08	-0.94	-
$TS^{(b13-b14)}$	-1.40	-0.52	1.92	-0.99	-

b14	-1.42	-0.46	1.88	-1.17	-
TS$^{(b14-b15)}$	-1.39	-0.44	1.82	-1.21	-
b15	-1.46	-0.41	1.87	-1.14	-
TS$^{(b15-b16)}$	-1.44	-0.03	1.47	-1.13	-
b16	-1.48	0.15	1.32	-1.23	-

Table S5. The relevant elementary reactions steps of ammonia synthesis reaction on $Ru_3@Mo_2CO_2$ in the optimal associative alternative pathway I and dissociation pathways. for microkinetic analysis using CatMAP software package.

Step	alternative I	Step	dissociation
R1	*_s + N2_g –> NN*	R1	*_s + N2_g –> NN*
R2	NN* + H2_g –> NN&HH*	R2	NN* <–> N−N* –> N&N*
R3	NN&HH* <–> NN&H−H* –> NN&H&H*	R3	N&N* + H2_g –> N&N&HH*
R4	NN&H&H* <–> NN&&H−H* –> NN&&&HH*	R4	N&N&HH* <–> N&N&H−H* –> NHN&H*
R5	NN&&&HH* <–> N&HN−H* –> N&HNH*	R5	NHN&H* <–> NHN−H* –> NHNH*
R6	N&HNH* <–> N&H&N−H* –> N&H&NH*	R6	NHNH* + H2_g –> NHNH&HH*
R7	N&H&NH* <–> N&H&&N−H* –> N&H&&NH*	R7	NHNH&HH* <–> NHNH−H&H* –> NHHNH&H*
R8	N&H&&NH* <–> N&H&&&N−H* –> N&H&&&NH*	R8	NHHNH&H* <–> NHHNH−H* –> NHHNHH*
R9	N&H&&&NH* +H2_g –> NHNHHH*	R9	NHHNHH* + H2_g –> NHHNHH&HH*
R10	NHNHHH* <–> NHNH−HH* –> NHNHH&H*	R10	NHHNHH&HH* <–> NHHNHH−HH* –> NHHNHHH&H*
R11	NHNHH&H* <–> NH−NHH&H* –> NH&NHH&H*	R11	NHHNHHH&H* –> NH3_g + NHH&H*
R12	NH&NHH&H* <–> NH&NHH−H* –> NH&NHHH*	R12	NHH&H* <–> NHH−H* –> NHH&&H*
R13	NH&NHHH* –> NH* + NH3_g	R13	NHH&&H* <–> NH&H−H* –> NHHH*

R14	NH* +H2_g −> NH&HH*	R14	NHHH* −> NH3_g + *_s
R15	NH&HH* <−> NH−HH* −> NHH&H*		
R16	NHH&H* <−> NHH−H* −> N&HHH*		
R17	N&HHH* <−> NH&H−H* −> NHHH*		
R18	NHHH* −> NH3_g + *_s		

Table S6. Calculated turnover frequencies (TOFs) of the ammonia synthesis reaction on $Ru_3@Mo_2CO_2$.

P = 100 bar	TOF /s^{-1}sit ×10^{-1}		log(TOF) /s^{-1}sit ×10^{-1}	
T /K	alternative I	dissociation	alternative I	dissociation
300	1.63×10^{-16}	1.09×10^{-50}	-15.79	-49.96
350	1.34×10^{-12}	9.45×10^{-41}	-11.87	-40.02
400	5.67×10^{-10}	2.61×10^{-33}	-9.25	-32.58
450	6.08×10^{-8}	1.56×10^{-27}	-7.22	-26.81
500	2.52×10^{-6}	6.37×10^{-23}	-5.60	-22.20
550	5.23×10^{-5}	3.69×10^{-19}	-4.28	-18.43
600	6.48×10^{-4}	4.94×10^{-16}	-3.19	-15.31
650	5.36×10^{-3}	2.12×10^{-13}	-2.27	-12.67
700	3.15×10^{-2}	3.49×10^{-11}	-1.50	-10.46
750	1.34×10^{-1}	2.01×10^{-9}	-0.87	-8.70
800	4.03×10^{-1}	0	-0.40	--
850	8.59×10^{-1}	0	-0.07	--
900	1.35	0	0.13	--
950	1.60	0	0.20	--
1000	1.15	0	0.06	--
1050	0	0	--	--
P = 48 bar	TOF /s^{-1}sit ×10^{-1}		log(TOF) /s^{-1}sit ×10^{-1}	
T /K	alternative I	dissociation	alternative I	dissociation
300	1.15×10^{-16}	2.28×10^{-41}	-15.94	-40.64
350	6.49×10^{-13}	3.08×10^{-32}	-12.19	-31.51
400	2.73×10^{-10}	2.37×10^{-25}	-9.56	-24.62
450	2.92×10^{-8}	5.78×10^{-20}	-7.53	-19.24
500	1.21×10^{-6}	1.25×10^{-15}	-5.92	-14.90
550	2.51×10^{-5}	4.64×10^{-12}	-4.60	-11.33

600	3.10×10^{-4}	4.51×10^{-9}	-3.51	-8.35
650	2.52×10^{-3}	1.46×10^{-6}	-2.60	-5.83
700	1.45×10^{-2}	1.37×10^{-4}	-1.84	-3.86
750	5.38×10^{-2}	0	-1.27	--
800	1.35×10^{-1}	0	-0.87	--
850	2.26×10^{-1}	0	-0.65	--
900	2.38×10^{-1}	0	-0.62	--
950	3.61×10^{-3}	0	-2.44	--
1000	0	0	--	--
1050	0	0	--	--
T = 610 K	TOF $/s^{-1}sit\times10^{-1}$		log(TOF) $/s^{-1}sit\times10^{-1}$	
P = /bar	alternative I	dissociation	alternative I	dissociation
1	5.15×10^{-6}	0	-5.29	--
7.6	7.24×10^{-5}	1.19×10^{-8}	-4.14	-7.92
14.2	1.40×10^{-4}	4.01×10^{-8}	-3.85	-7.40
20.8	2.08×10^{-4}	3.23×10^{-8}	-3.68	-7.49
27.4	2.75×10^{-4}	2.60×10^{-8}	-3.56	-7.59
34	3.43×10^{-4}	2.15×10^{-8}	-3.46	-7.67
40.6	4.10×10^{-4}	1.82×10^{-8}	-3.39	-7.74
47.2	4.78×10^{-4}	1.58×10^{-8}	-3.32	-7.80
53.8	5.46×10^{-4}	1.40×10^{-8}	-3.26	-7.86
60.4	6.13×10^{-4}	1.25×10^{-8}	-3.21	-7.90
67	6.81×10^{-4}	1.13×10^{-8}	-3.17	-7.95
73.6	7.48×10^{-4}	1.03×10^{-8}	-3.13	-7.99
80.2	8.16×10^{-4}	9.46×10^{-9}	-3.09	-8.02
86.8	8.83×10^{-4}	8.75×10^{-9}	-3.05	-8.06
93.4	9.51×10^{-4}	8.14×10^{-9}	-3.02	-8.09
100	1.02×10^{-3}	7.61×10^{-9}	-2.99	-8.12

T = 700 K	TOF /s^{-1}sit ×10^{-1}		log(TOF) /s^{-1}sit ×10^{-1}	
P = /bar	alternative I	dissociation	alternative I	dissociation
1	3.30×10^{-4}	0	-3.48	--
7.6	1.24×10^{-3}	0	-2.91	--
14.2	3.18×10^{-3}	0	-2.50	--
20.8	5.25×10^{-3}	0	-2.28	--
27.4	7.38×10^{-3}	0	-2.13	--
35	9.86×10-3	3.39×10^{-5}	-2.01	-4.47
40.6	1.17×10^{-2}	1.08×10^{-4}	-1.93	-3.97
47.2	1.39×10^{-2}	1.35×10^{-4}	-1.86	-3.87
53.8	1.61×10^{-2}	1.43×10^{-4}	-1.79	-3.84
60.4	1.83×10^{-2}	1.43×10^{-4}	-1.74	-3.84
67	2.05×10^{-2}	1.39×10^{-4}	-1.69	-3.86
73.6	2.27×10^{-2}	1.33×10^{-4}	-1.64	-3.88
80.2	2.49×10^{-2}	1.27×10^{-4}	-1.60	-3.90
86.8	2.71×10^{-2}	1.21×10^{-4}	-1.57	-3.92
93.4	2.93×10^{-2}	1.15×10^{-4}	-1.53	-3.94
100	3.15×10^{-2}	1.09×10^{-4}	-1.50	-3.96
